# A BCI Based Alerting System for Attention Recovery of UAV Operators

**DOI:** 10.3390/s21072447

**Published:** 2021-04-02

**Authors:** Jonghyuk Park, Jonghun Park, Dongmin Shin, Yerim Choi

**Affiliations:** 1 Department of Industrial Engineering and Institute for Industrial Systems Innovation, Seoul National University, 1 Gwanak-ro, Gwanak-gu, Seoul 08826, Korea; chico2121@snu.ac.kr (J.P.); jonghun@snu.ac.kr (J.P.); 2 ai.m Inc., Gangnamdae-ro, Gangnam-gu, Seoul 06241, Korea; 3 Department of Industrial and Management Engineering, Hanyang University, 55 Hanyangdaehak-ro, Sangnok-gu, Ansan-si 15588, Korea; dmshin@hanyang.ac.kr; 4 Department of Data Science, Seoul Women’s University, Hwarang-ro, Nowon-gu, Seoul 01797, Korea

**Keywords:** brain computer interaction, unmanned aerial vehicle, EEG-signal, attention recovery, alerting system, graphical user interface

## Abstract

As unmanned aerial vehicles have become popular, the number of accidents caused by an operator’s inattention have increased. To prevent such accidents, the operator should maintain an attention status. However, limited research has been conducted on the brain-computer interface (BCI)-based system with an alerting module for the operator’s attention recovery of unmanned aerial vehicles. Therefore, we introduce a detection and alerting system that prevents an unmanned aerial vehicle operator from falling into inattention status by using the operator’s electroencephalogram signal. The proposed system consists of the following three components: a signal processing module, which collects and preprocesses an electroencephalogram signal of an operator, an inattention detection module, which determines whether an inattention status occurred based on the preprocessed signal, and, lastly, an alert providing module that presents stimulus to an operator when inattention is detected. As a result of evaluating the performance with a real-world dataset, it was shown that the proposed system successfully contributed to the recovery of operator attention in the evaluating dataset, although statistical significance could not be established due to the small number of subjects.

## 1. Introduction

A brain computer interface (BCI) is a system that allows direct communication between human brain and external devices by translating brain signals into commands [1,2]. After demonstrating that people’s mental status can be observed and adjusted by means of BCI, the coverage of BCI application has expanded nowadays. Specifically, inattention, fatigue, and drowsiness detection became significant subjects since those mental status are deeply associated with accidents in diverse areas [3,4]. Among them, inattention, which is a mental status of decreased attention, arouses wide concern in both academia and industry due to many catastrophic accidents reported to be caused by the inattention [5].

In particular, as the number of accidents has increased with the introduction of unmanned aerial vehicles (UAVs), inattention detection has become an important research area. There are three major factors that are likely to cause a UAV operator’s loss of his/her attention: first, the most significant factor is the separation between cockpit and vehicle [6]. Second, operators of UAVs are required to perform monotonous and routine tasks. Lastly, operating UAVs is highly attention-demanding work due to the complexity of instrumentations and continuous interaction with air traffic management system on the ground [7,8].

Meanwhile, neurocognitive states related to inattention that cause operator performance degradation are identified as four categories: mind wandering, effort withdrawal, perseveration, inattentional blindness & deafness [9]. Effort withdrawal and mind wandering may occur at a low level of task engagement, whereas perseveration and inattentional blindness & deafness occur at high level of task engagement [10]. Arousal is also a factor that contributes to performance degradation resulting from degraded mental state [9] and has been reported to cause inattentional phenomena or effort withdrawal [11,12,13,14]. We focus on the mind wandering status among the four neurocognitive states, and propose an alert system that can effectively perform attention recovery using visual and auditory stimulus.

The proposed system, based on BCI, is composed of inattention detection and alert providing modules to automatically detect inattention of a UAV operator and timely provide alerts on the basis of detection. To achieve this, we utilize the operator’s electroencephalogram (EEG) signal that is the recording of human brain electrical activity by using electrodes placed on his/her scalp [15].

EEG-signal is known to be rapidly affected by attention fluctuations [16], while it can be acquired in a non-invasive manner. This suggests that the proposed system is able to detect inattention and alert UAV operators with minimal disturbance on the operators. Furthermore, the signal can be collected and recorded continuously which is important due to the constant fluctuations of attention status.

Many studies have been attempted to detect the mental state of a driver or pilot using EEG [17,18,19,20,21,22]. In the previous study, mental states, such as distraction, workload, and fatigue, were classified or detected by methods, such as support vector machine (SVM) [23], linear discriminant analysis (LDA), and neural networks. There have also been studies to improve the detection performance by combining other signals, such as functional near-infrared spectroscopy (fNIR), eye tracking measurements, and electrocardiogram (ECG) to the EEG [24,25,26,27,28]. However, since these studies focused on detecting the mental states, and did not proposed an alert system for real-world applications. In this study, for real-world application, the system that can detect the inattention state and alert the pilot effectively is developed.

The proposed system is composed of three components, signal processing, inattention detection, and alert providing modules. The signal processing module continuously collects and records the EEG-signal in real-time, and it preprocesses the signal to be applicable for the detection module. In the detection module, a detection model trained by using the preprocessed signal data detects the inattention of a UAV operator. When an operator’s inattention is detected by the detection module, the alert providing module generates alerts for the operator to help the recovery of his/her attention.

Specifically, the signal processing module acquires performance values of flights, velocity and altitude, in addition to the EEG-signal to monitor an operator’s attention status as initial inputs for the proposed system. Adaptive auto-regressive (AAR) and principal component analysis (PCA) coefficients are calculated for the vectorization and dimension reduction of the collected EEG-signal in the signal processing module. Then, hidden Markov models (HMMs) are employed as an inattention detection model. Finally, the alert providing module generates visual and auditory stimulus with an LED lamp, screen, and speaker. To validate the effectiveness of proposed system, two experiments on inattention detection and attention recovery using real-world datasets are designed and performed.

This paper is organized as follows. First, related works are briefly introduced in Section 2. Then, the proposed system and its validation experiments are provided in Section 3 and Section 4, respectively. In Section 5, the proposed system and the limitation of the study are discussed. Lastly, the paper is concluded in Section 6.

## 2. Related Work

### 2.1. Brain Computer Interaction

BCI is an interface that directly connects the human brain to external devices by analyzing brain signals, such as EEG-signal [29]. It has been investigated since the 1970s [30,31] and considered as a promising interface over the last three decades [32]. More promising and productive BCI research has been conducted with the advancement of signal processing methods and the emergence of effective types of equipment treating brain signals [2,33].

In the early days, most BCI applications focused on providing a new non-muscular communication channel for patients who suffer from severe neuromuscular disorders, such as amyotrophic lateral sclerosis and spinal cord injury [34,35,36]. Furthermore, BCIs have been considered as alternatives for restoring mobility for the above-mentioned people. Specifically, many researchers have investigated to control the movement of computer cursor [34,37], to manipulate the movement of robot [38], and to display the user’s intended words to the computer screen [39,40].

Recently, after it was proved that interpreting brain signals could provide useful information about the mental and emotional states, the coverage of BCI was expanded. For example, Choi et al. [41] proposed the HMM-based detection method to identify the attention level of a UAV operator using the EEG-signal. This study is supported by Makeig and Jung [42] presenting that the EEG-signal was substantially accurate measures for inattention generating significant variations in EEG-signal. Most current studies on BCI use the EEG-signal as a tool to infer mental status [43,44]. The EEG-signal has been introduced to the various domain, including a monitoring system for healthcare and investigative tools for human cognition and behavior [45,46]. Furthermore, Chae et al. [47] attempted to manipulate the movement of a robot by utilizing the EGG-signal.

### 2.2. Inattention Detection and Alerting System

There are a vast amount of studies on the inattention detection of drivers or pilots using diverse measures, such as subjective, vehicle-based, behavioral, and physiological measures [48]. Subjective measures indicate the result of a survey, which is hard to be utilized in a real-time system, and vehicle-based and behavioral measures capture the event that occurred due to an operator’s inattention state. For instance, when a driver is constantly crossing a red line, it can be inferred that the operator might lose his/her attention. Lastly, the physiological measure is a record of biological signals, such as EEG-signal, which begins to be widely utilized for the detection of mental states of a person as it becomes much easier to acquire due to the advancement of signal acquisition device technology.

Unlike studies on the inattention detection, only a few investigated the development of a real-time inattention detection system with an alerting module for attention recovery. Ha et al. [49] focused on the comparison of attention recovery results according to the type of stimulus, and Niu et al. [50] explored verbal and visual stimulus to find the most effective one for fighter pilots.

Meanwhile, there were studies for the system of attention recovery, including inattention detection and alerting modules using facial information. A system proposed by Vyas et al. [51] first extracts the face region and facial features. The system detects fatigue based on the extracted features and subsequently generates alert signals, using LEDs and buzzers. Similarly, Awasekar et al. [52] detects the face of a driver first, and then eyes are traced from the captured face image, which is used to identify if the driver is yawning or nodding. However, above methods have a limitation that they can be influenced by factors other than attention. In other words, the change in the facial expression might be a response to another external stimulus. Furthermore, time delays are inevitable to detect inattention since additional time is required to capture the face of the driver accurately and process the image data [53].

Recent work by Awasekar et al. [52] utilized EEG-signal for the inattention detection in the attention recovery system with an alerting module. However, it adopted basic statistics for the detection of inattention, resulting in inaccurate detection. Particularly, EEG-signal is high dimensional and sequential data which is hard to handle, and advanced approaches are required for the detection.

In summary, although a number of studies were conducted on the development of inattention detection method or the discovery of the most effective stimulus, only a few were interested in implementing a BCI system with an alerting module for attention recovery. Research on the attention recovery system has limitations in terms of measures and methods they adopted. Moreover, little research has been carried out on the systems for UAVs.

### 2.3. EEG-Signal in Inattention State

Many studies have been conducted on how the EEG signal changes in the inattention state. In particular, there have been attempts to determine the inattention state by representing the EEG-signal in delta, theta, alpha, and beta frequency bands and using values corresponding to each band. However, only theta band’s amplitude was addressed to be consistently elevated in the inattention state [54,55,56,57,58,59], and the directions of change for delta, alpha, and beta in the inattention state were not consistent in the previous studies. Some studies reported an increase in the delta [55,58] and alpha [54,56,59,60] bands’ amplitude, while others reported no change in the delta [56,59] and alpha [58] bands’ amplitude in the inattention state. Moreover, several studies presented that a decrease in the amplitude of the alpha [57] or beta [57,59] band occurs. Each study claimed different aspects, except for theta, so we examine whether our inattention determination method is suitable by measuring theta’s changing pattern in the inattention state.

## 3. Proposed Attention Recovery System

### 3.1. System Overview

**Figure 1:**
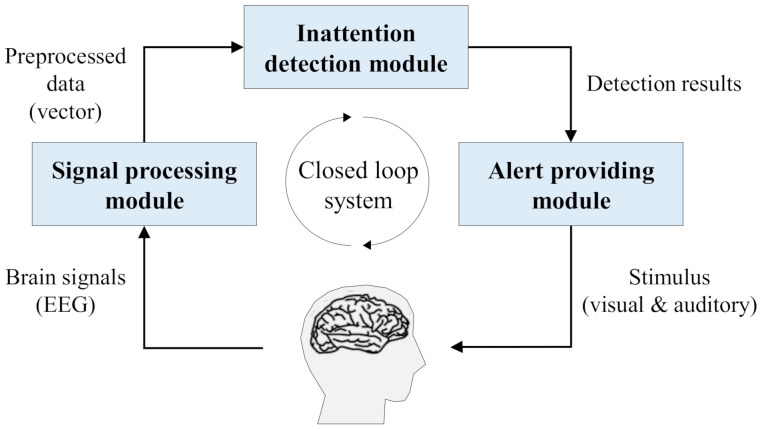
Overview of the proposed attention recovery system for unmanned aerial vehicle (UAV) operators.

The proposed system is designed to help an operator to recover his/her attention by continuously monitoring his/her attention status and timely providing alerts. The system consists of three modules, including signal processing, inattention detection, and alert providing modules. Figure 1 describes the overall process of the proposed attention recovery system, where its initial input is an operator’s brainwaves, and the final output is a stimulus for the operator from the alert providing module. It employs EEG-signal as an indicator of attention status, and visual and auditory stimuli are used for alerting operators to recover their attention.

Specifically, the signal processing module acquires EEG-signal of an operator maneuvering a flight, and it concurrently collects the velocity and altitude of the flight, called performance values. Then, the module extracts features of the collected EEG-signal to build a vector to meet the input shape of the following detection module by using AAR and PCA. In the inattention detection module, machine learning models are trained and utilized to detect the inattention of the operator using the preprocessed data. In this process, performance values are used only for the model training, and the trained model receives a preprocessed EEG-signal as a sole input for the detection. Lastly, the alert providing module decides whether to generate a stimulus to help the operator recover his/her attention according to the results from the detection module. For instance, when the inattention of an operator is detected, the alert providing module is activated, and visual and auditory stimuli are provided simultaneously. The following describes the constitutions of each module and its operating procedures.

### 3.2. Signal Processing Module

The signal processing module is composed of three steps, data acquisition, preprocessing, and inattention labeling. EEG-signal and two performance values, velocity and altitude, are collected in the data acquisition step. The collected data is then preprocessed to meet the input shape of the following inattention detection module. Particularly, for the training of the inattention detection model, the occurrences of an operator’s inattention during a flight are marked by analyzing the performance values in the inattention labeling step.

#### 3.2.1. Data Acquisition Step

EEG-signal is recorded to monitor fluctuations in the attention status of an operator, and the velocity and altitude of the flight that the operator is maneuvering are acquired for the training of inattention detection models which classify an input EEG-signal into two attention status, attention and inattention. We assume that the acquired EEG-signal could be classified into one of them.

**Figure 2:**
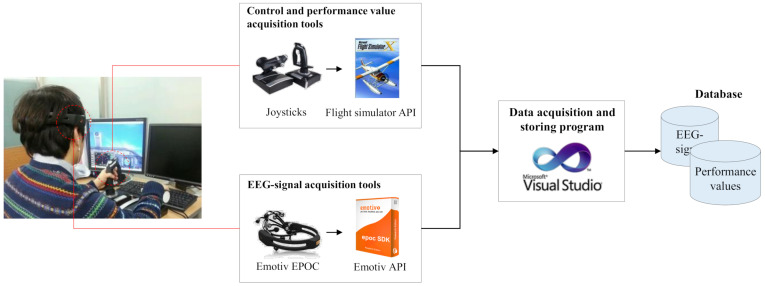
Details of data acquisition step in signal processing module.

Figure 2 describes the details of the data acquisition step and its output. The data acquisition step utilizes two kinds of input devices, Emotiv EPOC [61] and joysticks. Emotiv EPOC is a commercial device for recording EEG-signal, and it supports wireless connection via Bluetooth communication. Joysticks are used to maneuver a flight in a simulator, and flight simulator API [62] enables the acquisition of sequences of altitude and velocity throughout the maneuvering. Note that the performance values are used as criteria for the attention status of operators with an assumption that the emergence of high variance in velocity or altitude is an indicator of a flight failure due to an operator’s inattention.

**Figure 3:**
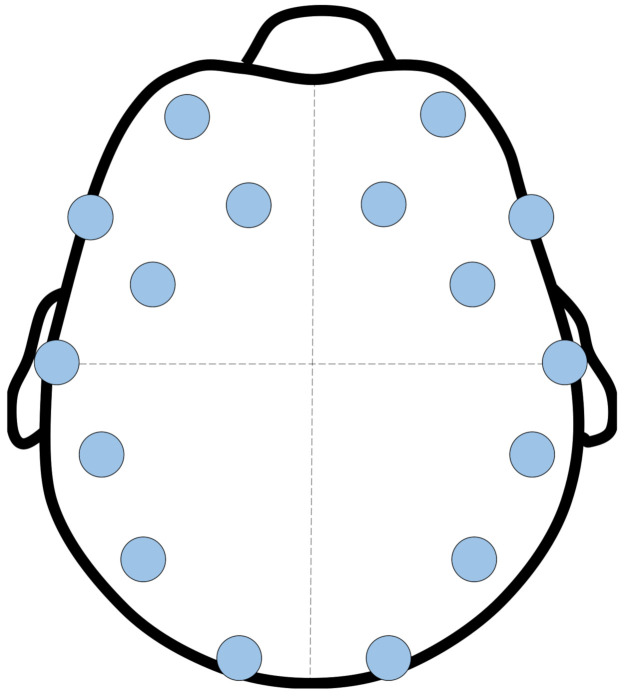
Fourteen channels for electroencephalogram (EEG)-signal acquisition using Emotiv EPOC.

Specifically, Emotiv EPOC has a form of headset with 14 channels that individually collect EEG-signal at a regular interval. Each channel indicates a position in the scalp of an operator for collecting EEG-signal. Specific positions of the 14 channels in Emotiv EPOC are shown in Figure 3. By using Emotiv EPOC API, EEG-signal is collected at a frequency of 6 Hz and from 14 channels according to the international 10–20 electrode standard. Then, it filtered by band pass filter with bandwidth from 2 to 42 Hz and decomposed into amplitude (in micro-volts) of four frequency bands (delta, theta, alpha, and beta) through fast Fourier transformation (FFT). Before the feature extraction, the amplitudes were averaged across the 14 channels and normalized according to each band.

To this end, the acquired an EEG-signal has a total of 56 properties, collected from 14 channels and four frequency bands per each channel, and it is stored in a database. In addition, the performance values are recorded at the same interval as that of collecting EEG-signal, and they are also stored in the database.

#### 3.2.2. Data Preprocessing Step

In order to transform the collected EEG-signal to fit the input format of the inattention detection model and to find the most meaningful features for the inattention detection, the data preprocessing step is performed. The data preprocessing step includes three activities, noise removal, feature extraction, and dimensionality reduction. The following explains the activities in detail.

At first, noises appearing in raw EEG-signal and performance values are removed. Since the sequences of velocity and altitude of a flight fluctuate largely at the beginning of maneuvering, it is hard to determine whether those fluctuations indicate an inattention of an operator. Therefore, the beginning part of the recorded EEG-signal and performance values are removed prior to annotating the attention or inattention. After the removal, each time interval of a maneuvering is marked as attention or inattention based on the variances in sequences of performance values.

Then, meaningful features are extracted from the EEG-signal which is composed of 56 properties. Such high dimensionality of features increases the complexity of classification and lowers its computational speed. Furthermore, it is widely accepted that the classification accuracy is degraded when utilizing all features compared to when using selected meaningful features [63,64]. Moreover, EEG-signal reacts sensitively to changes of operators other than their attention status, such as a muscle movement. Therefore, it is important to utilize features that are in a lower dimension and are more robust to subtle changes than the original data. For these reasons, feature selection or dimension reduction is essential for BCI system designs [65].

**Figure 4:**
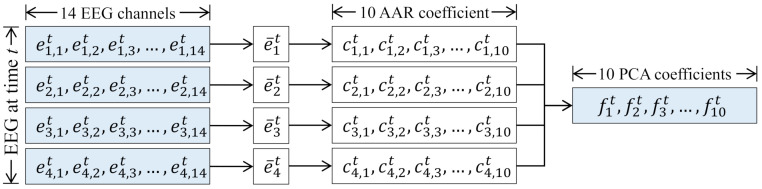
Overall process of data preprocessing step.

To this end, we adopt AAR coefficients and PCA for the feature extraction and dimensionality reduction, respectively. The overall process is depicted in Figure 4. AAR coefficients are widely used for the feature extraction of sequential data [66,67], and they were firstly adopted to EEG-signal classification problem in Reference [68]. AAR makes an adaptive model to fit data segments, and the model’s coefficients are estimated by using least square methods [67].

In addition, PCA is one of the most popular dimensionality reduction methods, which finds principal components that are linearly uncorrelated from the original data [69]. Since PCA reduces the dimension of feature space, while focusing on particular features and ignoring others, it selects informative features for classification, leading to an improvement of classification performances. The research carried out by Subasi and Gursoy [70] demonstrated that applying PCA achieves much higher classification accuracy than considering all features. Based on the findings of the research, PCA was adopted for dimension reduction in diverse applications [71,72,73].

For simplicity, we denote the EEG-signal after the noise removal as a three-dimensional tensor *E* which is a sequence of a matrix at time *t* denoted by $e^{t}$, where t=1,⋯,T. $e^{t}$ is composed of $e_{i,j}^{t}$ where i=1,⋯,4 indicate frequency bands, delta, theta, alpha, and beta, respectively, and j=1,⋯,14 is the corresponding 14 channels.

We firstly average $e_{i,j}^{t}$ across *j*, resulting in ${\bar{e}}_{i}^{t}$, in order to obtain a single value for each frequency band. By analyzing ${\bar{e}}_{i}^{t}$ for t=1,⋯,T, the predefined number of AAR coefficients are calculated and utilized as features, denoted by $c_{i,k}^{t}$, $k=1,\cdots ,n_{c}$, where $n_{c}$ is the predefined number of AAR coefficients. Lastly, among $c_{i,k}^{t}\forall i,k$, the predefined number of features are selected by using PCA, building a feature vector at *t* denoted by $f_{t}$ which is a vector of $f_{l}^{t}$, $l=1,\cdots ,n_{p}$, where $n_{p}$ is the predefined number of features.

#### 3.2.3. Inattention Labeling Step

To train an inattention detection model, $f_{t}$ needs to be labeled as attention or inattention. We assumed that $f_{t}$ during a failure flight indicates the occurrence of an operator’s inattention status, based on the finding that the occurrence of inattention leads to a performance degradation [74,75]. During an unstable flight, where there exist large fluctuations in performance values, $f_{t}$ is labeled as an inattention, and it is labeled as an attention during a stable flight, where performance values are successfully kept at a constant level.

We denote altitude and velocity as $a_{t}$ and $v_{t}$ for t=1,⋯,T, respectively. According to the variance of values of $a_{t}$ and $v_{t}$ for a certain time window *w*, the attention level of an operator at *t*, denoted as $l_{t}$, is determined. When the operator is determined to be attentive, the value of 1 is assigned to $l_{t}$, and the value of 0 is assigned to it, otherwise. Sub-sequences of EEG-signal, where large fluctuations of performance values exist, were labeled as inattention. Specifically, the time window whose variance of the two performance values was above the 50th percentile was judged to be inattention. However, the number of inattention occurrences was too low, so that the number of sampling was doubled for the inattention status.

### 3.3. Inattention Detection Module

**Figure 5:**
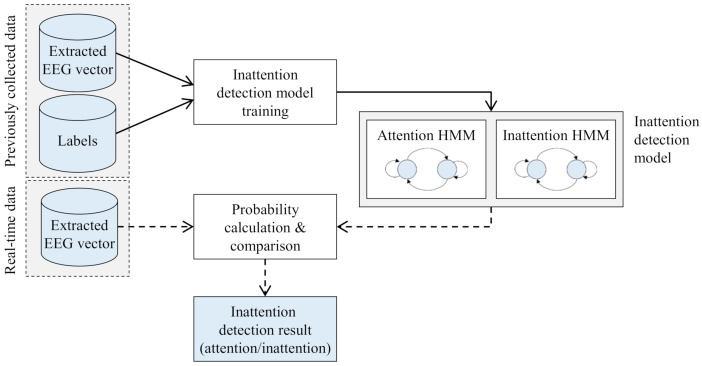
Overview of the inattention detection module using hidden Markov models (HMM).

In the inattention detection module, the preprocessed EEG-signal and performance values are utilized for training and testing an inattention detection model, which classifies $f_{t}$ into attention or inattention. Figure 5 describes the overall process of inattention detection. First of all, a model is trained using the previously collected data composed of extracted EEG vectors and corresponding attention labels. When an operator begins a flight, extracted EEG vectors are continuously generated and fed into the trained inattention detection model in real-time. Then, the attention level of the operator is determined by the model.

A machine learning methodology is utilized for the detection model to recognize the inattention automatically and maximize detection performances by analyzing patterns inherent in the previously collected EEG-signal. Meanwhile, the model has subject-dependent property, which means that an operator has its own detection model that has been trained by using his/her data since each individual shows distinct patterns in EEG-signal [76]. Note that since we attempt to build a subject-dependent model causing the insufficient of data, it is impossible to utilize the deep learning method, which performs appropriately with sufficiently large-sized data.

Among diverse machine learning models, we adopt one of the most well-known machine learning models for sequential data, HMM. Since EEG-signal is non-stationary and contains time information [69], a model for classifying EEG-signal is supposed to deal with the sequence of feature vectors extracted from the acquired EEG-signal and to catch time information intrinsic in EEG-signal. HMM is one of the simplest Bayesian classifiers generating the probability of observing a given sequence of feature vectors [77] and is reported to be effective in detecting non-stationary changes of EEG-signal [78,79].

HMM-based inattention detection model consists of two HMMs, attention HMM and inattention HMM. Each HMM is trained by using the corresponding sequences of EEG-signal. For instance, attention HMM is trained by using the sequences of an operator’s EEG-signal, which were generated when the operator is in an attention state.

### 3.4. Alert Providing Module

**Figure 6:**
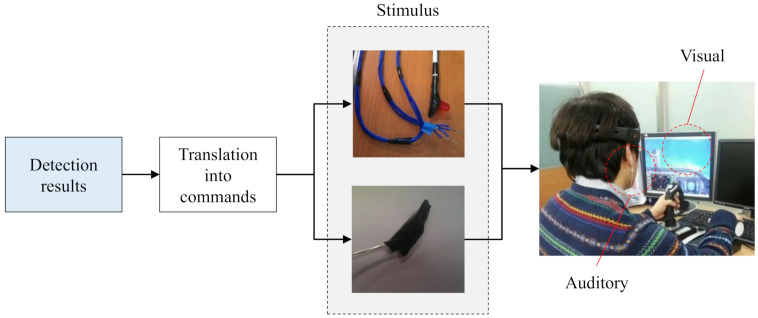
Overview of the alert providing module.

Alert providing module is activated when an operator is determined to be not focused from the previous inattention detection module. As shown in Figure 6, it includes a command converter that translates the inattention detection result of an operator into a command and a stimuli generator that provides stimuli to the operator according to the command. First, the inattention detecting result of an operator who is currently maneuvering a flight is converted to a command that triggers the stimuli generator. There exist two commands, alert and stay, according to the detection results. Then, the command activates the stimulus generator when the command is alert, while the generator continues to wait for the activation command when the command is stay. Note whether the generator is activated or not, other modules, signal processing and inattention detection modules, run continuously for the real-time monitoring of the operator’s status.

Every stimulus has an unique effect on the mental or biological status of operators. For instance, it is well-known that people are more responsive to an auditory stimulus than a visual one [80]. This is the reason that the most alarms are provided with auditory stimulus. Therefore, to develop an effective alerting system for UAVs based on EEG-signal, it is important to find the most effective stimulus (or stimuli) that helps operators recover their attention.

Niu et al. [50] explored the behavior and mental patterns of fighter pilots with visual and auditory stimuli. It is reported that the auditory stimulus in the form of slow verbal communication was optimal as it achieved the best performances in the conducted experiments, causing the earlier attention. Moreover, Reference [50] demonstrated that performances achieved by utilizing both visual and auditory stimulus together were the best. Thus, we provide both auditory and visual stimulus simultaneously in the alert providing module.

### 3.5. Implementations

**Figure 7:**
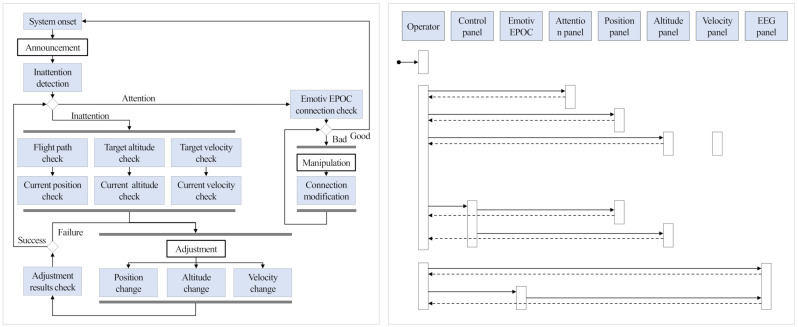
User sequence diagram (**right**) and flow chart (**left**) of the proposed attention recovery system.

By exploiting the findings mentioned in the previous section, we implemented the attention recovery system for UAV operators. Figure 7 shows the user sequence diagram and flow chart of the proposed system. During a flight, an operator’s attention level is continuously monitored by analyzing EEG-signal acquired from the operator in real-time. When inattention of the operator is detected, an alarm is provided to the operator, and the operator is requested to examine and adjust flight location and performance values. In addition, the system checks the connection with the EEG-signal acquisition device every cycle, and, when a poor connection is detected, a manual adjustment is requested. After the adjustments, the system keeps monitoring the attention level of the operator.

**Figure 8:**
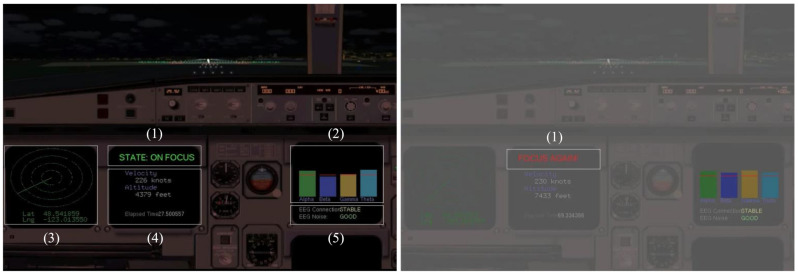
Snapshots of graphical user interface (GUI) in the proposed brain computer interface (BCI)-based attention recovery system during attention state (**left**) and inattention state (**right**).

We also implemented a graphical user interface (GUI), where an operator can monitor his/her flight situation, and an alert is presented to the operator when inattention is detected. Figure 8 shows the GUI of the proposed BCI-based attention recovery system. The left image in Figure 8 is the snapshot of the proposed GUI when an operator is in an attention state, where (1) shows the attention state of an operator, (2) depicts the amplitude of the collected EEG signal’s four frequency bands, (3) and (4) illustrate the current flight situation, and (5) shows the connection status with an EEG-acquisition device. The right image in Figure 8 shows the GUI when an operator is in an inattention state, and the GUI flickers with alarming sounds.

## 4. Experiment

In order to investigate the effectiveness of the proposed system, two types of test were designed and conducted using a real-world dataset. In the first test, we evaluated the accuracy of the HMM-based inattention detection model, and the effectiveness of the alert providing module in recovering attention status was validated in the second test.

### 4.1. Experiment Settings

#### 4.1.1. Data Acquisition Procedure

Three male and one female subjects aged between 22 and 28 participated in the experiments as summarized in Table 1. All subjects had enough intellectual ability to understand and carry out the experiments and did not have any physiological or neurological disorder. Before the experiments, subjects answered a brief questionnaire on their states, and none of them reported any discomfort or any kind of fatigue.

**Figure 9:**
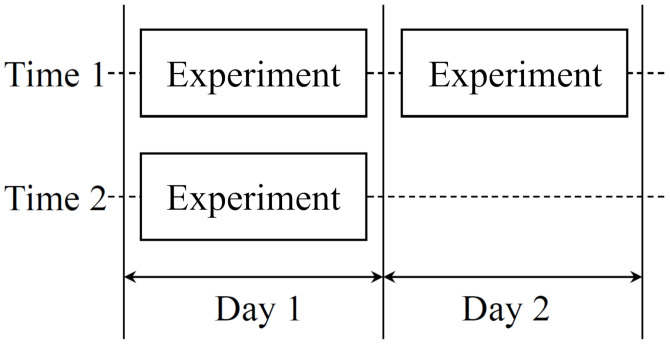
Schedule of the three data acquisition experiments.

The data acquisition were performed through the following procedure. Before conducting full-scale experiments, subjects were trained for more than thirty minutes to get accustomed to the simulator and equipment. Maneuvering of the selected path was repeatedly performed for three times over two days as the schedule shown in Figure 9. Sufficient intervals between experiments were provided to prevent the effect of tiredness.

**Figure 10:**
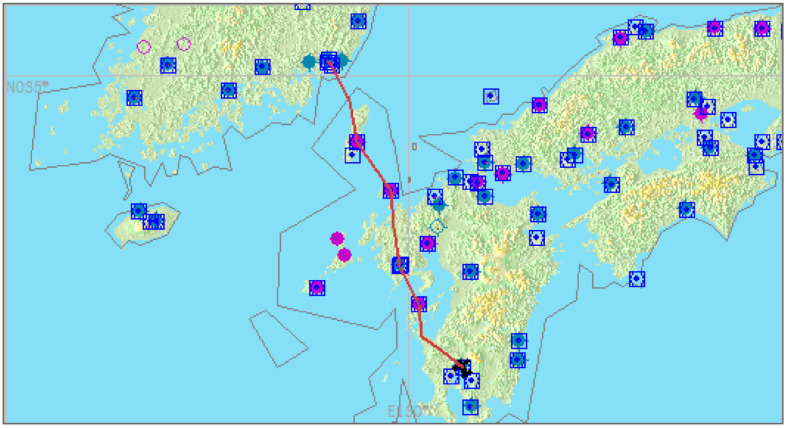
Example of a flight path utilized in the experiments.

We tried five different scenarios and evaluated their difficulty by surveying the subjects after the test flight. Finally, the medium difficulty (level 2) scenario was chosen for two reasons. First, since too-difficult tasks affect flight performances, our approach cannot be applied. Second, the too-easy task does not require any attention, so subjects tend to commit too few mistakes during the flight. Details of the scenario are as follows. The path is depicted in Figure 10, which has four waypoints but a mostly straight flight path from Kagoshima to Kimhae. Furthermore, we asked subjects to follow the instructions. They are requested to follow a given path under a deviation threshold of 0.005 rad and maintain altitude and velocity of 6500 feet and 250 knots, respectively.

Each maneuvering lasted around twenty to thirty minutes, and EEG-signal of subjects and performance values of a flight during the maneuvering were collected every 1/6 s (6 Hz). Minor artifact rejection was done by visual inspection on the collected EEG-signal. In addition, EEG-signal acquired at the early stage of maneuvering was removed as there exists a tendency of EEG-signal being rapidly fluctuated at the beginning of a flight.

Around 2487 s of EEG-signal was recorded, and a total of 14,860 instances were sampled for the performance comparison experiments. Since it is sampled at 6 Hz for 10 s, the window length is 60 points. Additionally, as we allowed 10% overlap, the step size would be 6 points. Then, the instances were randomly split into training and test data at a ratio of 8:2. In other words, 11,744 and 2936 instances were used for training and test, respectively. Both the number of AAR coefficients, $n_{c}$, and the number of PCA features, $n_{f}$, were fixed to 10.

#### 4.1.2. Evaluation Measures

For the performance evaluation, we utilized well-known measures in classification problems, accuracy, recall, and precision. Accuracy is the ratio of the number of correctly detected cases over all cases and is calculated by using Equation (1).

(1) $$Accuracy=\frac{TP+TN}{TP+FP+FN+TN}.$$

TP and TN indicate the numbers of cases that are correctly predicted as attention and inattention, respectively, while FN and FP are the numbers of cases that are actually attention but detected as inattention and are actually inattention but detected as attention, respectively, as shown in Table 2.

Moreover, recall is the ratio of the number of cases correctly detected as an inattention over the total number of inattention cases defined as Equation (2).

(2) $$Recall=\frac{TP}{TP+FN}.$$

Precision is the ratio of the number of cases correctly detected as an inattention over the total number of cases detected as inattention defined as Equation (3).

(3) $$Precision=\frac{TP}{TP+FP}.$$

#### 4.1.3. Experimental Details

**Figure 11:**
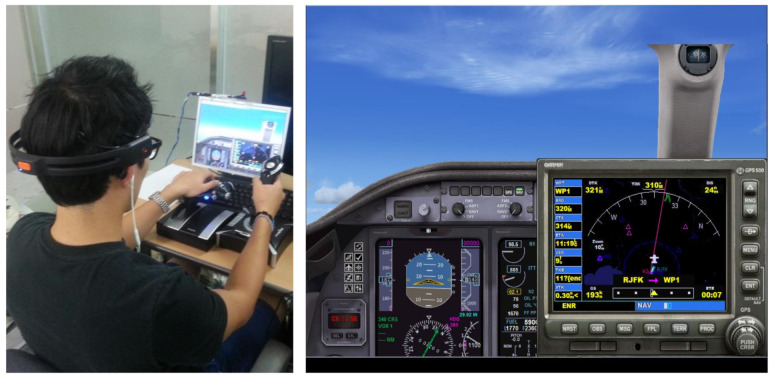
Snapshots of experimental environment including equipment (**left**) and flight simulator (**right**).

The experimental environment is depicted in Figure 11, where a subject operates a flight simulator (shown in the right image) using joysticks while wearing an EEG-signal acquisition device (shown in the left image). For the acquisition of EEG-signal, we utilized a non-invasive device, Emotiv EPOC manufactured by Emotiv Corporation, as mentioned in Section 3.2.1. The device collects EEG-signal of an operator from 14 channels, AF3, AF4, F3, F4, F7, F8, FC5, FC6, P7, P8, T7, T8, O1, and O2. Emotiv EPOC API enables the real-time acquisition of EEG-signal from a device to a database.

In addition, for the maneuvering, Microsoft Flight Simulator X was employed. Subjects can experience tasks similar to those of UAV as the simulator provides realistic maneuvering situations with diverse types of aircraft. The simulator was operated using two kinds of joysticks, one for controlling altitude and direction and the other for velocity. Flight information, such as altitude, velocity, latitude, longitude, pitch, bank, yaw, and deviation, from the given path of an aircraft were recorded by using SimConnect API. Particularly, altitude and velocity of flights were collected with EEG-signal of subjects.

Meanwhile, the HMM-based inattention detection model was implemented using the RHmm package of R programming language. As the paper is focused on proposing a framework rather than the detection model’s performance, we did not optimize the hyper-parameters of methods. We utilized the default parameter provided by package: Three hidden states and random initialization of probability. Details on the HMM-based inattention detection can be found in Reference [41].

In order to compare the performances of the proposed HMM-based inattention detection model, we employed SVM. Among the traditional shallow learning models, SVM is known to show the best performance attributed to its strength in finding the optimal generalization error bound. Similar to the HMM-based inattention detection model, we used the default parameters provided by the SVM function in R project library, e1071, with a radial kernel.

### 4.2. Experiment Results

In this section, we present four types of experimental results. First of all, we explore the inattention labeling results of the data preprocessing module, where an operator’s attention or inattention during maneuvering is determined by analyzing the performance values of a flight. After that, we evaluate whether it is appropriate to determine the inattention from velocity and altitude by investigating EEG-signal frequency band’s change pattern for all subjects. Then, the performance of the inattention detection module is described and compared with that of SVM-based inattention detection. Finally, the effectiveness of alert providing module is validated by comparing the performance values before and after the providing alerts.

**Figure 12:**
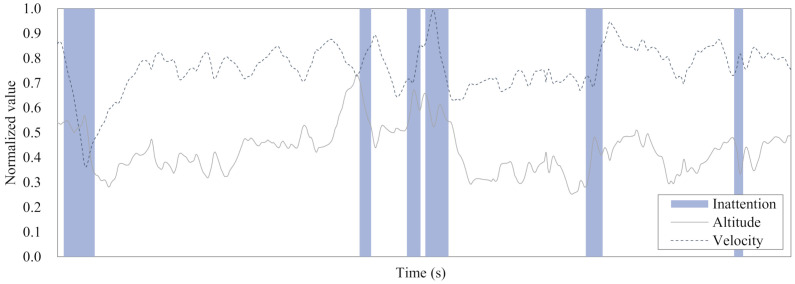
Example of labeled data according to the variances of altitude and velocity. Blue colored box indicates the inattention of an operator.

First, Figure 12 shows a result of the attention labeling. Time windows colored in blue indicate the inattention of an operator. The ratio of inattention status of the utilized dataset was 14.32% after removal of the fluctuations at the beginning of flight, while it was 19.58% among the whole maneuvering. The ratio remained around 40% until 10 min of the early phase during the total of 30 min flight.

**Figure 13:**
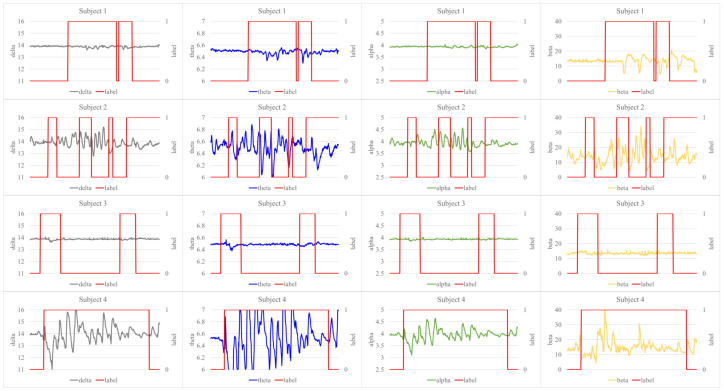
Graphs between the amplitudes of EEG-signal’s four frequency bands and the inattention labels obtaining from each subject during flight. Each row represents the subject, and each column represents the frequency band of the EEG-signal.

Second, the amplitudes of EEG-signal’s four frequency bands for each subject are depicted in Figure 13. The red line represents the inattention label, where value 1 indicates the inattention state. It can be shown that the inattention labels determined from the velocity and altitude are most closely related to the increase in the amplitude of the theta band value among other frequency bands.

Third, we performed the inattention detection performance comparison with SVM-based model, and the results are shown in Table 3. HMM outperformed SVM in terms of accuracy and precision, while SVM worked slightly better than HMM in terms of recall. The superior performance of HMM to SVM indicates that the consideration of sequential patterns in EEG-signal was effective. Particularly, HMM showed much higher precision, about 24% enhanced, than SVM with a similar level of recall. It implies that there exist cases that cannot be judged by using static information only, but sequential information became a hint for the detection as expected.

Lastly, we verify the effectiveness of providing auditory and visual stimulus to operators for recovering attention. Basic settings for the experiment are the same as the previous detection experiment, and data collection and analysis were performed identically. Visual alert stimuli was presented using both LED lamp and screen with flickering and auditory stimuli was expressed as a beeping sound through the speaker when the inattention was detected.

**Figure 14:**
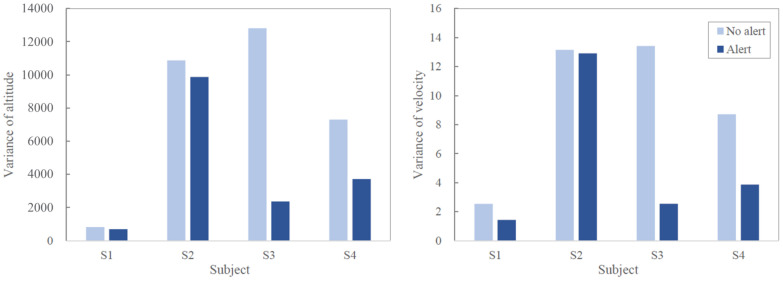
Variances of two performance values, altitude (**left**) and velocity (**right**), for two situations, when alert is provided or not.

The variances of two performance values, altitude and velocity, are compared based on whether the alert is provided when the operator’s inattention is detected. The results of these experiments are shown in Figure 14. From the results, it can be concluded that the proposed system was successful in helping operators to recover their attention. Providing alert was effective for subjects as we closely investigated the attention states for cases where inattention occurs, and then alert is provided.

## 5. Discussion

In this section, we first analyze why the HMM-based model has superior performance in inattention detection over the SVM-based model. Next, the reason why the performance improvement of subject 2 in Figure 14 is small is investigated. Finally, the limitations of this study are discussed, and further research directions related to the limitations are suggested.

The EEG-signal we used is sequential data. Since the information of the previous time step can be usefully utilized to determine the attention or inattention of the next time step, HMM-based model, which has strength in modeling sequential data, outperformed SVM-based model. Furthermore, SVM-based model has a disadvantage of being sensitive to noise [81]. As mentioned earlier, a noise removal step is involved in the data preprocessing process, but this is limited to the beginning part of the recorded EEG-signal. Since EEG-signal can generate noise due to various reasons [82], this noise may be reflected in the hyperplane construction of SVM, causing incorrect classification. Meanwhile, the performance of subject 2 was less improved than others, and the effectiveness of the alert system in terms of velocity and altitude was not well demonstrated. Subject 2’s overall ability to maneuver a flight is not good, as shown in Figure 14, so we guess that the response to the alert of subject 2 is insignificant than others.

This study has several limitations. The first limitation is that statistical significance of the proposed architecture cannot be determined because the number of subjects is insufficient. As mentioned above, we conducted the questionnaire about their states before conducting the experiment. More than four subjects were actually recruited, but only those who had no problem participating in the experiment were selected through rigorous screening. Besides, the schedule of performing repeated experiments on one subject and the long total time for the entire experimental stages were also factors that hindered data collection from more subjects. In such a process, the number of subjects decreased. The effect could be confirmed for the subjects who participated, but statistically, it was not enough. Therefore, it is a future work to assess the performance of the proposed architecture with the data collected from more subjects. The second limitation is that a consumer-grade EEG device was utilized for the experiment. Commercial EEG acquisition devices are universal, but strong impedance variations can occur, which sometimes reduce the quality of the data. Thus, evaluating with a high-performance EEG acquisition system is another future work. Finally, the flight scenario was so easy and short that the pilot might have maintained high attention level. Through various experimental scenarios, including longer flight paths and frequent flight direction changes, we will see if our system is an effective countermeasure even in situations that cause stronger attentional impairment.

## 6. Conclusions

This paper presented a novel attention recovery system based on BCI by automatically detecting an UAV operator’s inattention and providing alert signals. The proposed system utilizes EEG-signal to generate data for the inattention detection module using HMM. When an operator’s inattention is detected, the alert providing module generates visual and auditory stimuli to recover the operator’s attention. The experiments using a real-world dataset collected from subjects during maneuvering of a flight simulator demonstrated that the proposed system successfully detected the inattention of the subjects and helped them to recover attention, although statistical significance could not be established due to the small number of subjects.

Therefore, we plan to evaluate the system from more recruited subjects. At this time, it is expected to improve the performance by including an advanced alert providing module that utilizes diverse stimuli, such as tactile and human voice. Furthermore, developing an effective labeling method is another future work to obtain high-quality training data for the inattention detection module.

## Figures and Tables

**Table 1:** Summary of the subjects participated in experiments in terms of gender and age.

Subject	Gender	Age
S1	male	26
S2	male	22
S3	male	24
S4	female	28

**Table 2:** Confusion matrix for inattention detection, where TP, FN, FP, and TN indicate true positive, false negative, false positive, and true negative, respectively.

		Detected	
		Inattention	Attention
Actual	Inattention	TP	FN
	Attention	FP	TN

**Table 3:** Results of the inattention detection experiments in terms of three evaluation measures, accuracy, precision, and recall.

ML Classifier	Accuracy	Precision	Recall
HMM	0.766	0.879	0.674
SVM	0.734	0.709	0.683

## Data Availability

Not applicable.
